# Targeted Radiation and Immune Therapies—Advances and Opportunities for the Treatment of Prostate Cancer

**DOI:** 10.3390/pharmaceutics15010252

**Published:** 2023-01-11

**Authors:** Anusha Muralidhar, Hemanth K. Potluri, Tanya Jaiswal, Douglas G. McNeel

**Affiliations:** 1 University of Wisconsin Carbone Cancer Center, 1111 Highland Avenue, Madison, WI 53705, USA; 2 7007 Wisconsin Institutes for Medical Research, 1111 Highland Avenue, Madison, WI 53705, USA

**Keywords:** combination therapy, radiation therapy, targeted radionuclide therapy, immunotherapy, prostate cancer

## Abstract

Prostate cancer is the most diagnosed malignancy in men in the United States and the second leading cause of cancer-related death. For localized disease, radiation therapy is a standard treatment that is often curative. For metastatic disease, radiation therapy has been primarily used for palliation, however, several newer systemic radiation therapies have been demonstrated to significantly improve patient outcomes and improve survival. In particular, several targeted radionuclide therapies have been approved for the treatment of advanced-stage cancer, including strontium-89, samarium-153, and radium-223 for bone-metastatic disease, and lutetium-177-labeled PSMA-617 for patients with prostate-specific membrane antigen (PSMA)-expressing metastatic castration-resistant prostate cancer (mCRPC). Contrarily, immune-based treatments have generally demonstrated little activity in advanced prostate cancer, with the exception of the autologous cellular vaccine, sipuleucel-T. This has been attributed to the presence of an immune-suppressive prostate cancer microenvironment. The ability of radiation therapy to not only eradicate tumor cells but also potentially other immune-regulatory cells within the tumor immune microenvironment suggests that targeted radionuclide therapies may be well poised to combine with immune-targeted therapies to eliminate prostate cancer metastases more effectively. This review provides an overview of the recent advances of targeted radiation agents currently approved for prostate cancer, and those being investigated in combination with immunotherapy, and discusses the challenges as well as the opportunities in this field.

## 1. Introduction to Prostate Cancer Radiotherapy

Prostate cancer is the most diagnosed cancer among men with an estimated 268,490 new cases and 34,500 deaths in the United States in 2022 [1]. Traditional treatments for localized disease include surgery or radiation therapy, and with or without limited androgen deprivation therapy [2]. Although a majority of patients respond to these therapies, about one third of them have recurrence. Recurrent disease is generally treated with androgen deprivation, however, tumors ultimately develop castration resistance, and the median life expectancy of metastatic, castration-resistant prostate cancer (mCRPC) is less than three years [3].

Radiation therapy that is used in both localized and advanced stage disease can be categorized into three main categories: external-beam radiation therapy (EBRT), which uses an X-ray machine (linear accelerator) to produce high-energy photons outside the body that target cancer cells; brachytherapy (BT), which uses radioactive seeds administered internally, and targeted radionuclide therapy (TRT) which involves administering radionuclides coupled to cancer targeting compounds to irradiate tumor cells.

## 2. External Beam Radiation Therapy

EBRT is a potentially curative treatment for localized prostate cancer, and typically prioritized for patients who are not candidates for prostatectomy and/or have adverse pathologic features [4,5,6]. The standard regimen for treating low-risk disease is 75–80 Gy, and for intermediate-risk and high-risk prostate cancer, EBRT is often combined with short-term androgen deprivation [7,8].

During the past three decades, technological advances have greatly improved radiation therapy delivery with emphasis on improving target definition with smaller radiation fields [9]. To achieve dose escalation, 3-dimensional conformal radiation therapy (3D-CRT) was developed using planning computed tomography (CT) scans, and as reported in the RTOG 9406, phase I/II trial, yielded favorable outcomes for localized prostate cancer [10]. With the development of more sophisticated treatment-planning software in the mid-1990s, intensity-modulated radiation therapy (IMRT) emerged as an advanced form of conformal therapy. In addition to IMRT, image-guided radiotherapy (IGRT) is an essential adjunct for dealing with daily changes to the target anatomy [11]. It has been reported that IMRT may be more effective than 3D-CRT for higher dose treatments [12].

Several studies have indicated that prostate cancer responds well to EBRT delivered in fewer fractions with higher doses (hypofractionation) [13,14,15]. Stereotactic body radiation therapy (SBRT), also called stereotactic ablative radiotherapy (SABR), is a technique that can deliver higher doses in hypofractionated regimens and has shown comparable biochemical control and morbidity to standard fractionation schedules in prospective randomized trials [13,16]. Results with SBRT in limited numbers of patients with low-risk prostate cancer suggest that this approach is feasible, with comparable efficacy and safety to other forms of radiation therapy [17,18,19].

In contrast to conventional photon (X-ray) therapies, proton beam therapy (PBT) can also confer several advantages in terms of tumor targeting and dose exposure. Multi-institutional randomized trials (NCT01617161, NCT03561220) are comparing IMRT versus PBT for impacts on treatment-related toxicity and quality of life, however, no prospective trials have evaluated the efficacy of PBT relative to EBRT [20].

## 3. Brachytherapy

In the early 19th century, Pasteau and DeGrais explored treatments for prostate cancer by implanting radium sources through a catheter in the urethra or rectum. This initial approach led to improvement in techniques and instruments for brachytherapy using radioactive sources by multiple others [21]. In particular, the interest in brachytherapy was revived by Bagshaw and colleagues in the late 1950s with a series of patients being cured by high-energy cobalt treatment [22]. In the early 1980s, Whitmore experimented with a variety of different radioisotopes implanted as permanent brachytherapy sources in patients, and found this approach was well tolerated [23]. Holm and colleagues applied transrectal ultrasound to the guidance of iodine-125 seed placement [24]. Iodine-125, palladium-103, and cesium-131 are now the three isotopes routinely used for low-dose-rate (LDR) prostate brachytherapy [25]. Neither isotope appears to be more effective than another based on the available data [26]. In some cases, LDR brachytherapy is used in combination with EBRT in men with intermediate- or high-risk prostate cancer [27]. Escalating the dose of radiation through high-dose-rate (HDR) brachytherapy with iridium-192 has also been used as a monotherapy and/or in combination with EBRT, and this has been a promising approach for treating locally advanced prostate cancers [28,29,30,31].

There have been no randomized prospective trials to compare the efficacy of EBRT and BT treatments. However, a meta-analysis of randomized clinical trials provided evidence to support the use of a BT boost after EBRT to improve biochemical progression-free survival (bPFS) in intermediate- and high-risk patients [32].

## 4. Targeted Radionuclide Therapy

Despite potentially curative therapy for localized prostate cancer, nearly one third of patients will ultimately progress to metastatic disease [33,34,35]. Due to the inability to feasibly target all metastatic lesions, EBRT has typically been used only for palliative purposes in this setting [36,37]. However, in recent years, TRT has emerged as a promising treatment strategy for metastatic prostate cancer. Strontium-89 (Sr-89) choloride and samarium-153-ethylene-diamino-tetramethylene-phosphonate (Sm-153-EDTMP) were the first TRT agents approved for palliative use due to their ability to target metastatic bone disease, although they did not improve overall survival [38,39]. Radium-223 dichloride, which is a calcium mimetic, similarly targets bone metastatic disease, and was the first alpha-emitting radionuclide approved by FDA as a treatment for mCRPC based on evidence of extended survival [40,41].

While these TRT agents have been useful for patients with disease localized exclusively to the bone, they are not effective for those with other disease sites. Hence, other investigations have focused on compounds that specifically target cancer cells rather than the bone. One of the most studied targets for these approaches has been prostate-specific membrane antigen (PSMA) which is highly expressed on prostate cancer cells [42]. As the first PSMA-targeting antibody, capromab pendetide was initially used as an imaging tool for prostate cancer. Labeling of this agent with therapeutic isotypes demonstrated limited clinical efficacy, probably because it binds to an intracellular epitope of PSMA, requiring the internal domain of PSMA to be exposed externally [43]. However, J591, a monoclonal antibody that binds the extracellular domain of PSMA, has been conjugated to multiple radionuclides and evaluated in trials for patients with mCRPC. Using an activated monoclonal antibody-fluorophore conjugate of J591, Nakajima and colleagues successfully imaged prostate cancer in vivo in murine prostate tumor models [44]. Bander et al. conducted several trials assessing dose-limiting toxicities and efficacy of radiolabeled J591 and demonstrated that the agent can accurately target metastatic prostate cancer sites in bone and soft tissue, and may be useful for targeting diagnostic or therapeutic imaging agents [45,46]. In particular, a phase II clinical trial evaluating a single administration of ^177^Lu-J591 found this to be well tolerated and capable of targeting metastatic sites. Of 47 patients with mCRPC treated, 59.6% experienced a decrease in PSA, and 1 out of 12 with measurable disease achieved a partial response. Those with low PSMA expression by imaging were less likely to respond [47]. As a mixed alpha and beta emitter, bismuth-213 (^213^Bi) has also demonstrated preclinical activity when tagged to the PSMA-targeting antibody J591 [48,49,50,51]. However, ^213^Bi-J591 has not yet been evaluated in patients with prostate cancer.

More recently, a small molecule ligand that binds PSMA, PSMA-617, has been radiolabeled with different radionuclides and investigated for both diagnostic imaging and therapeutics (theranostic agent). ^68^Ga-PSMA-617 was the first approach to utilize PSMA-617 for the diagnosis of prostate cancer. As reported by Liu and others, PET/CT imaging of ^68^Ga-PSMA-617 was useful as an imaging biomarker to assess metastatic disease, not detectable by conventional imaging, to stratify the risk of metastatic prostate cancer [52,53,54]. Preclinical studies of PSMA-617 with the ^177^Lu beta emitting radioligand yielded encouraging results for safety and efficacy as reported by Ruigroket et al. [55]. ^177^Lu-PSMA-617 (Lu-PSMA) was evaluated in a prospective single-arm phase II study in which 57% of patients experienced PSA declines greater than 50%. Patients also experienced pain improvements, and median PFS and OS of 7.6 months and 13.5 months, respectively, were identified [56]. This was confirmed in a randomized phase III trial in which patients with mCRPC, who had received prior taxane chemotherapy and at least one AR pathway inhibitor agent, were randomized to receive ^177^Lu-PSMA-617 or a non-chemotherapy standard of care (SOC). This trial demonstrated that Lu-PSMA therapy was effective in generating a longer radiological progression-free survival (rPFS, 8.7 months) in patients with PSMA+ mCRPC who previously received standard of care therapies, as well as overall survival (15.3 months), compared to 3.4 and 11.3 months, respectively, for the SOC group. These findings led to FDA approval in 2022 for the use of Lu-PSMA as a treatment for mCRPC following previous treatment with chemotherapy and a second-generation AR pathway targeting agent [57,58].

PSMA-617 has also been evaluated conjugated to other radioisotopes, notably with the higher energy alpha emitter actinium-225 (Ac-225). A series of small studies have evaluated ^225^Ac-PSMA-617 in patients with mCRPC, including patients resistant to ^177^Lu-PSMA-617 [59,60,61,62]. A retrospective meta-analysis of 6 trials comprising 201 patients treated, showed that PSA declines > 50% occurred in 66.1% of patients, with low rates of hematological toxicity. The most common adverse effect, as with Lu-PSMA, was xerostomia, which occurred in 77.1% of all patients. Further prospective clinical trials with this agent are anticipated [63]. A list of radionuclides commonly used for brachytherapy and targeted radionuclide therapy of prostate cancer is shown in Table 1.

## 5. Immunotherapy in Prostate Cancer

The concept of immunotherapy for prostate cancer dates back to the 1970s when Ablin and colleagues reported anecdotal cases of metastatic prostate cancers that regressed after being treated locally with cryotherapy [64]. The identification of autologous antibodies that recognized the prostate tissue suggested that this abscopal-type treatment response was immune mediated [65]. This led to significant interest in developing immune-based strategies for prostate cancer treatment. Early efforts focused on vaccines aimed at generating T-cell and antibody responses to the prostate. This concept was ideal for prostate cancer since the prostate is a non-essential organ, and therefore an immune response directed against the prostate need not be necessarily limited to malignant cells. Since then, there have been several approaches to developing anti-tumor vaccines, most commonly delivering inactivated or modified tumor cells (antigen non-specific), or delivering components of tumors to stimulate immune responses to specific antigenic targets (antigen-specific vaccines) [66]. Five phase III clinical trials have been conducted in patients with mCRPC using different vaccine approaches. Four of these approaches did not demonstrate improved overall survival [67,68,69,70,71]. To date, only sipuleucel-T met its primary endpoint of extending survival in patients with advanced metastatic prostate cancer and was approved by FDA as a treatment for mCRPC in 2010 [71]. Sipuleucel-T is comprised of autologous antigen-presenting cells loaded ex vivo with a fusion protein of a tissue-specific antigen (prostatic acid phosphatase, PAP) and GM-CSF. The approval of sipuleucel-T has demonstrated that vaccine therapies can be useful in the treatment of prostate cancer, however, the absence of significant objective radiographic responses or substantial changes in serum prostate specific antigen (PSA) with sipuleucel-T, or other vaccines when used as monotherapies, has led to the exploration of vaccines as parts of combination therapies.

Since 2010, the success of immune checkpoint blockade therapies for multiple cancer types has led to their exploration in the treatment of prostate cancer as well. Single-agent trials using agents blocking either CTLA-4 or PD-1 have advanced through clinical testing and have also been evaluated in randomized phase III clinical trials. Unfortunately, the results of these trials have been relatively disappointing. In particular, two phase III trials using CTLA-4 blockade (ipilimumab), either alone or combined with bone metastasis-targeted radiation therapy, did not demonstrate prolonged overall survival [72,73]. Clinical trials using PD-1/L1 blocking agents have similarly demonstrated little evidence of single-agent activity in earlier phase clinical trials for patients with metastatic prostate cancer [74,75]. A combined PD-1 and CTLA-4 blockade demonstrated slightly more clinical activity against advanced prostate cancer, but with significantly more toxicity, hence is not being further pursued [76]. A phase III trial using enzalutamide with or without atezolizumab found no difference in overall survival [77]. Similarly, a recently completed phase III trial using docetaxel with or without pembrolizumab (KEYNOTE-921) for patients with advanced prostate cancer showed no improvement in overall survival. Notwithstanding, earlier phase clinical trials have suggested that checkpoint blockade therapies may have efficacy in combination with other agents, including vaccines, and hence combination trials are being further pursued [78,79].

More recently, efforts have been directed toward recruiting effector T cells to the sites of tumor with the development of bispecific T-cell engagers (BiTE). These agents consist of a tumor-specific domain and a T-cell activating domain. Several such agents targeting PSMA are in clinical stages of development. In particular, pasotuxizumab (AMG 212) and acapatamab (AMG 160) have demonstrated objective responses in patients with mCRPC [80,81]. A phase I clinical trial with JNJ-081, a similar bispecific antibody for PSMA and CD3, demonstrated transient reductions in PSA in patients with mCRPC [53]. A second generation BiTE approach, HPN424, has also recently completed evaluation in a phase I/II clinical trial [53]. More BiTE approaches targeting other prostate-specific surface molecules are being explored.

Although all of the vaccine approaches evaluated in phase III trials were effective in activating antigen-specific T cells, most did not improve clinical outcomes when used alone in treating advanced prostate cancer. Similarly, despite immune checkpoint blockade therapy being effective for many different cancer types, these treatments have been relatively ineffective as monotherapies for advanced prostate cancer. Together, these findings suggest that mechanisms of immune suppression are active in prostate cancer. Prostate cancer is known to have an immunologically “cold” tumor microenvironment characterized by a low mutational burden and limited infiltration of effector CD8+ T cells. In addition, the secretion of immune-suppressive cytokines like TGF-β and CXCR2 by prostate tumor cells leads to the recruitment of immunosuppressive populations, such as regulatory T cells (Treg) and myeloid-derived suppressor cells (MDSC), that have also been associated with poor clinical outcomes [82,83]. The expression of nitrous oxide synthase and indoleamine-2,3-dioxygenase by these populations can further suppress the activity of CD8+ T cells [83,84]. Consequently, these immunotherapeutic approaches may be most active when used in combination with agents that target the mechanisms that underlie tumor-associated resistance and immune evasion.

## 6. Rationale for Combining Radiation Therapy and Immunotherapy

Radiation therapy (RT) has effects on both tumor cells and immune cells within the tumor microenvironment, potentially making immunotherapies more effective against poorly immunogenic tumors. The optimal dose and fractionation of radiotherapy are likely important and may differ for each tumor type and immune microenvironment. In addition to its direct effect on cell killing, radiation at optimal doses can cause release of proinflammatory cytokines such as IL-1β and TNFα [85,86], increase FAS expression on tumor cells rendering them susceptible to FAS-ligand-expressing lymphocytes [87], and increase MHC-I expression on tumor cells making them more readily detected by CD8+ T cells [88]. In addition, RT-induced dsDNA fragments can be sensed by cGAS within irradiated cells in the tumor, activating STING signaling, and leading to a type I IFN response and increased cross-presentation by DCs. Radiation therapy can also increase CD86 and CD70 expression on intratumoral DCs, as well as promote DC maturation, increased expression of MHC II, and migration to the tumor-draining lymph nodes to present antigens to CD8+ T cells [89,90]. Additionally, as the dose of radiation increases, greater accumulation of DNA damage occurs [91]. DNA damage leads to cell death, which is accompanied by release of damage-associated molecular patterns and a cascade of inflammatory response activation known as immunogenic cell death [92]. RT-induced immunogenic cell death can lead to the release of tumor-associated antigens that, cross-presented by activated DC, may elicit an adaptive immune response and lead to further increased T-cell infiltration into tumors [93].

In addition to the extensive literature demonstrating that RT has immunostimulatory effects, it has been shown that a significant portion of the anti-tumor effects of radiation are already mediated by immune populations. Immunocompromised mice require much higher doses of radiation to result in tumor eradication than do immunocompetent mice [94]. Additionally, when CD8+ T cells are depleted from tumor-bearing mice, the anti-tumor effect of radiation is greatly reduced [89]. Although RT initially results in temporary depletion of CD8+ T cells, this population is eventually activated and infiltrates the tumor, as reported in both preclinical models and human cancers [95,96,97]. It has also been shown that RT skews tumor-infiltrating CD8+ T-cell populations towards resident memory phenotypes, in part because these memory cells are more radioresistant [98,99]. There is also some evidence that RT can facilitate NK expansion, activation, and accumulation in draining lymph nodes, resulting in improved anti-tumor efficacy in vivo [100,101].

While rare, there have been reports of patients receiving radiation therapy who have had reduced tumor growth of lesions outside of the field of radiation, a process known as an abscopal effect. While poorly understood, the identification of anti-tumor responses to non-irradiated tumors, similar to what was observed following cryotherapy of prostate tumors [64], was assumed to be mediated by immune responses elicited to the tumor by the primary radiation therapy. Demaria and colleagues investigated this hypothesis in a murine model in which mice had syngeneic mammary tumors implanted on both flanks. Mice were then treated with or without flt3-ligand, and EBRT was delivered to one of the two tumors. They observed slower growth of the non-irradiated tumors only when both flt3-ligand and EBRT were both delivered, and this effect was eliminated if repeated using nude mice [102]. This effectively demonstrated that an abscopal effect could occur, and at least in this system, was mediated by T cells. The treatment effect using flt3-ligand suggested that increased antigen presentation by DC following radiation could be a potential mechanism for this response. Since the approval of several immune checkpoint inhibitors as treatments for cancer, abscopal responses have been observed with increasing frequency in patients treated with T-cell checkpoint inhibitors and radiation therapy [103]. This suggests that radiation therapy might not need to target all sites of tumor, at least for some tumor types, to be effectively combined with immunotherapy. The further study of the mechanism of abscopal responses could lead to new combination treatment approaches.

While conceptually radiation can be used to stimulate the development of an anti-tumor cytotoxic response, many tumors also have an increased number of immunosuppressive cell populations (tumor-associated macrophages (TAMs), MDSC, and Tregs) that are more radioresistant than the other immune subtypes. These populations can cause NK-cell and T-cell anergy and block DC maturation [104,105]. The dose of RT can therefore significantly affect the phenotype of immune cells and alter the signaling within the tumor microenvironment, making them pro- or anti-tumorigenic. For instance, RT can affect the functional polarization of macrophages that can activate either anti-tumor M1-like macrophages or immunosuppressive M2 macrophages, encouraging angiogenesis and remodeling of the stromal matrix to help tumor establishment [106]. However, high doses of RT have been correlated with increased TGFβ release that has potent immunosuppressive properties, including polarization of CD4+ T cells into Tregs, suggesting that targeted chemokine receptor antagonists might be effectively combined with RT [107,108]. Additionally, radiation can increase T-cell checkpoint molecule/ligand expression. Specifically, high-dose radiation results in upregulation of PD-L1 on tumor cells [109]. This explains the rationale behind treatment combinations with radiation and checkpoint blockade. As prostate cancer is associated with multiple immunosuppressive pathways, combining immunotherapy that targets these immunosuppressive pathways with radiotherapy may be a promising approach for increasing anti-tumor responses and preventing immune escape.

## 7. Preclinical Studies Combining Radiation Therapy and Immunotherapy

### 7.1. EBRT

The combination of radiation therapy and immunotherapy has shown promise in several preclinical studies of prostate cancer. In an early effort using an in situ gene therapy, HSV-tk (ADV-HSV-tk) in combination with ganciclovir therapy, and EBRT in the RM1/C56BL/6 murine prostate tumor model, it was shown that the combination group had the most significant inhibition of tumor growth and survival compared to either therapy alone, with a significantly higher frequency of infiltrating CD4+ T cells [110]. Similar results were found when adenoviral glioma pathogenesis-related protein 1 (AdGLIPR1) gene therapy and radiotherapy were combined in the 178-2 BMA/129/Sv murine model of metastatic prostate cancer. Combined treatment resulted in significant tumor growth suppression and survival extension compared with either therapy alone or control, and was associated with an increase in tumor-infiltrating CD4+ and CD8+ T cells [111].

In a study published by Harris et al., in the TRAMP mouse model in which the tumors expressed the hemagglutinin antigen (HA), a recombinant vaccinia virus vaccine targeting the hemagglutinin antigen (Vacc-HA) in combination with stereotactic radiation therapy with three doses of 10 Gy augmented CD4+ T-cell responses to the tumor vaccine [112]. In the same cancer model, Wada et al. demonstrated the combination of granulocyte macrophage-colony-stimulating factor (GM-CSF)-secreting cellular immunotherapy with stereotactic radiation of 12 Gy dose caused a dramatic increase of CD8+ T cells, resulting in long-term survival in 25% of the animals [113]. Similarly, in a preclinical study by Udayakumar and colleagues, EBRT of 5 Gy/day was administered for 5 days with intratumoral delivery of a vesicular stomatitis virus (VSV) expressing IFNβ. This led to an increase in systemic CD8+ T-cell numbers, eradication of tumors, and induced a memory response in 100% of animals when re-challenged with RM9 prostate tumors, suggesting the potential synergy between oncolytic viral therapies and RT [114].

Immune checkpoint blockade has also been evaluated in combination with radiation therapy in preclinical models. Dudzinski et al. reported a preclinical study in which mice implanted with MycCaP prostate cancer cells were treated with EBRT using 20 Gy × 2 in combination with anti-PD-1/L1. In this study, mice demonstrated a 70% longer survival with anti-PD-1 and 130% with anti-PD-L1, when delivered with EBRT, suggesting that EBRT can sensitize poorly immunogenic tumors to checkpoint blockade [115]. Similarly, Xu et al. demonstrated that blocking CSF-1R signaling enhanced the effectiveness of radiotherapy in a murine prostate cancer model by targeting the recruitment of tumor-infiltrating myeloid cells, illustrating the potential for clinical translation of targeting an immunosuppressive phenotype to enhance anti-tumor responses [104].

### 7.2. Brachytherapy

In spite of the fact that brachytherapy has been extensively used in the clinic and is well studied in a small number of preclinical xenograft models of prostate cancer, there are few reports of how this treatment might affect the tumor immune microenvironment. A recent preclinical report by Keom et al. evaluated a cohort of 28 patients who received high-dose BT for prostate cancer. They found that treatment induced the expression of several immune checkpoints (B7-H3, CTLA4, PDL1, and PDL2) within prostate tumors, suggesting the potential for combining BT and immune checkpoint blockade [116].

### 7.3. TRT

As a treatment option for metastatic prostate cancer, targeted radionuclide therapy offers the advantage of delivering a highly concentrated dose to multiple tumor sites while sparing the surrounding healthy tissue. In a preclinical study using C57BL/6 mice implanted with RM1-PGLS tumors expressing PSMA, anti-PD-1 in combination with ^225^Ac-PSMA-617 demonstrated improved tumor control, extended time to progression, and increased survival compared to monotherapies [117]. This was the first report demonstrating synergistic anti-tumor efficacy between PSMA-targeted TRT and PD-1 blockade in a syngeneic mouse model of prostate cancer.

NM600 is another TRT agent that has been investigated in preclinical models of prostate cancer. NM600 is an alkylphosphocholine (APC) developed based on investigations of phospholipid ethers that accumulate specifically in cancer cells instead of healthy cells [118]. The selective retention of NM600 by multiple tumor types, and its ability to be labeled with different radiometals without affecting tumor retention, has led to its exploration as a theranostic agent [119]. Potluri et al. demonstrated that ^90^Y-NM600 was selectively retained within TRAMP-C1 and Myc-CaP murine prostate tumors and, while it exhibited modest anti-tumor efficacy itself, treatment led to increased expression of PD-L1 by these tumors [120]. Combining PD-1 blockade with ^90^Y-NM600 led to an unexpected decrease in anti-tumor efficacy that was found to be due to increased activity of PD-1-expressing CD4+ Treg that persisted in tumors despite TRT treatment. These findings underscore the importance of understanding effects of TRT on the tumor microenvironment to guide effective combinatorial approaches [120].

## 8. Clinical Trials Combining Radiation Therapy and Immunotherapy

### 8.1. EBRT

Several clinical trials investigating the combination of radiation therapy and immunotherapy have been completed or are currently underway, as indicated in Table 2. Early trials evaluated EBRT in combination with tumor vaccines. Twardowski et al. reported a phase II clinical trial in which patients were randomized to receive sipuleucel-T with or without EBRT of 300 cGy/day to 3000 cGy total. In that trial, the EBRT was delivered to a single metastatic site, with treatment completed one week prior to sipuleucel-T infusion. The authors reported that radiation therapy did not enhance the generation of humoral or cellular responses following sipuleucel-T treatment, and no difference was noted in median PFS (2.46 months versus 3.65 months, *p* = 0.06) [121]. In another randomized phase II study, patients with localized prostate cancer were treated with definitive EBRT given with or without the Prostvac vaccine (priming immunization with a recombinant vaccinia virus encoding PSA followed by 7 booster immunizations with a recombinant fowlpox vaccine encoding PSA). EBRT was delivered between the fourth and sixth immunization. In 19 patients treated, the authors reported the generation of immune response to the PSA target protein in 13 patients, suggesting the feasibility of this approach, but there was no report of clinical efficacy [122].

EBRT has also been evaluated in combination with intratumoral delivery of immune-modulating treatments. In a small pilot trial of five HLAA2+ subjects with high-risk, localized prostate cancer, patients received a combination of androgen deprivation, 45 Gy of EBRT treatment, intraprostatic injection of autologous dendritic cells, and brachytherapy boost using either ^125^I or ^103^Pd seeds [123]. The EBRT was delivered in 25 fractions over five weeks, with 5 − 10 × 10^6^ dendritic cells injected after fractions 5, 15, and 25. Before and during treatment, serial prostate biopsies showed increased apoptotic cells and a parenchymal distribution of CD8+ cells. These findings, with the identification of IFNg-secreting ELISPOT response to defined prostate cancer antigens detected systemically, suggested this approach might be further developed to elicit therapeutic immune responses to prostate tumor antigens [123]. A phase III randomized, placebo-controlled trial is also currently underway testing intratumoral delivery of an oncolytic virus (ProstAtak, an adenoviral vector encoding thymidine kinase, given with oral valacyclovir), in patients with localized disease being treated with EBRT (NCT01436968) [127]. The primary endpoint for that trial is time to disease recurrence.

EBRT has also been explored in combination with immune checkpoint blockade. In a phase I/II dose-escalation trial by Slovin and colleagues, ipilimumab was given with or without EBRT to 75 patients with mCRPC; 8 Gy EBRT was delivered to 1–3 bone metastatic lesions 24–48 h prior to the first dose of ipilimumab. In that trial, the authors reported feasibility of this approach, and observed PSA reductions of ≥50% in 8/50 patients treated at the highest dose of ipilimumab, with one patient experiencing a complete response. This approach was further evaluated by Kwon et al. in a multicenter, randomized, double-blind phase III trial in which men with mCRPC and at least one bone metastasis received bone-directed EBRT followed by either ipilimumab or placebo. Although this study trended towards improvement in the combination arm, it did not reach statistical significance with a median OS 11.2 vs. 10.0 months (HR = 0.85; *p* = 0.053) [72].

### 8.2. TRT

It is conceivable that the studies described above using EBRT in patients with mCRPC were largely unsuccessful because only a small number of sites received radiation treatment, leaving other sites more resistant to the immune-based treatment. This has made the use of TRT to irradiate all metastatic sites simultaneously an appealing approach. In one of the first trials, 44 patients with mCRPC were randomized to receive ^153^Sm-EDTMP, a bone-targeting TRT agent, with or without PSA-TRICOM (Prostvac) vaccine [126]. The investigators reported that median PFS was longer in patients treated with the combination (3.7 months versus 1.7 months, *p* = 0.046), with no PSA declines in the ^153^Sm-EDTMP-alone arm, while 4 out of 21 (19%) had a 30% PSA decline in the combination group [126].

Two trials have been conducted using immunotherapy in combination with Ra-223. In a small, randomized phase II trial, 32 patients with mCRPC were randomized to receive sipuleucel-T treatment with or without six doses of monthly Ra-223, with sipuleucel-T intercalated between the second and fourth doses of Ra-223. The patients in the combination arm developed lower immune response to the vaccine antigen but experienced more PSA declines > 50% (31% vs. 0%, *p* = 0.04) and also demonstrated longer PFS (39 vs. 12 weeks) and OS (not reached vs. 2.6 years) [125]. Unfortunately, in the absence of a control group receiving radium-223, it is not clear if the clinical responses observed were improved with the addition of sipuleucel-T over Ra-223 alone. A separate phase Ib study evaluated atezolizumab and Ra-223 in 45 patients randomized to concurrent dosing or one of two staggered dosing schedules in which atezolizumab was started 28 or 56 days after the first dose of Ra-223. The median radiographic PFS was 3.0 months and median overall survival was 16.3 months. Overall, it was found that the combination elicited greater toxicity, regardless of the schedule of administration, with no clear evidence of additional clinical benefits for patients with mCRPC [124]. A separate phase II trial, randomizing patients with mCRPC to Ra-223 treatment with or without pembrolizumab, is currently underway (NCT03093428).

Since the approval of ^177^Lu-PSMA-617 in early 2022, several trials are underway using this agent in combination with immune checkpoint blockade, including pembrolizumab (NCT03658447, NCT03805594), or ipilimumab and nivolumab (NCT05150236).

## 9. Challenges and Opportunities

New insights into radiation biology are opening up new opportunities to better control cancer through rational combinations with biological therapies, including immune-based therapies. In recent years, the approval of several TRT approaches provides an opportunity to use radiation to treat metastatic disease, an approach that can be tailored to the individual patient and might thus be optimally combined with immune-based treatments. However, TRT approaches present challenges, including that there have been a limited number of dedicated high-energy accelerators to produce radionuclides. In addition, there have been hurdles in the development of good manufacturing practice guidelines for radiopharmaceutical production. Related to this, is that there are no guidelines on the use of individualized patient dosimetry. Currently, TRT is given with a fixed dosing regimen regardless of an individual patient’s tumor burden or tumor uptake as measured by a companion pretherapy PET tracer. This is despite evidence that more tailored therapy may improve outcomes. In murine prostate cancer models, Current et al. recently reported that intrasubject variability in PSMA expression, and the frequency of PSMA high-, medium-, or low-expressing cells, caused disparities in therapeutic effectiveness [128]. Further, this pretherapy dosimetry could be used to choose doses to reliably treat tumors and potentially spare organs at risk for toxicity [128]. Similar observations were made in patients as reported by Mannweiler and colleagues in which PSMA expression was highly heterogeneous in many primary prostate tumors (7/51) and metastases (6/51), and was absent in 2 primary and 8 metastatic tumors (<10%) [129]. In addition, the development of agents/schedules that result in synergistic anti-tumor activity without significantly increasing toxicity needs to be further investigated. For instance, in a recent retrospective study reported by Rosar et al., 15 patients with mCRPC treated with a combination of ^225^Ac-PSMA-617 and ^177^Lu-PSMA-617 experienced a prolonged PFS (9.1 months) and overall survival (14.8 months) with few adverse events [130]. The authors conclude that the use of different radionuclides with different half-lives and energy may limit toxicity and lead to greater efficacy. The incorporation of different radionuclides, dosing, and patient selection using companion diagnostics, will undoubtedly play a major role in treating specific tumor types. This may be particularly important if low doses of TRT treatment of human tumors can spare immunosuppressive regulatory T cells, or other immunosuppressive populations, and compromise the effects of immune-targeted therapy, as was observed in a preclinical study [120].

Combining targeted radiation and immunotherapy approaches is also still in early stages, and we lack adequate knowledge in several domains. For example, it is unclear if different radionuclide emitters have different effects on immunogenicity and if different radioisotopes affect immune populations within tumors in different ways. It thus remains to be determined which radioisotopes, or types of radiation, are best to combine with immunotherapy. There is also still much to learn about the role of immunotherapy sequencing and timing with respect to radiation. For example, if T-cell populations are eradicated within tumors shortly after radiation treatment, it may not be optimal to combine T-cell checkpoint blockade or T-cell activating therapies on the same days, but rather sequence these agents. This was demonstrated in a preclinical study in murine prostate tumors in which delivery of PD-L1 blockade with concurrent EBRT did not delay tumor growth despite an increase in PD-L1 expression on tumors following EBRT [131]. Similarly, it may be preferable to use radiation therapy after immune-based treatments to specifically ablate immunoregulatory populations such as Treg or MDSC that might be recruited by these immunotherapies.

## 10. Conclusions and Future Directions

Radiation therapy is a safe, effective, and commonly used treatment for prostate cancer. Through advancements in EBRT planning and delivery, the side effects of radiation treatment have been reduced and radiation doses to normal tissues have been decreased. The development of these technologies has enabled radiation dose escalation without causing an increase in toxicity. While these therapies have a clear role in localized prostate cancer, their use in metastatic disease has been for palliation only since they cannot be delivered to all sites of tumor. The use of TRT, however, provides an opportunity to treat all sites of disease, and the approvals of Ra-223 and ^177^Lu-PSMA-617 for mCRPC provide evidence that these approaches have a role in the treatment of metastatic disease. The ability of radiation to modulate the tumor immune microenvironment suggests that these therapies are well poised to be used in combination with immune-based therapies. Preclinical studies suggest this is a feasible approach, and in some cases have highlighted potential obstacles. Clinical trials are just beginning, but we expect over the next five years that there will be multiple clinical trials using different TRT agents, with different radionuclides that may confer different effects on the tumor immune microenvironment. We further expect these to be used in combination with different immune targeted therapies, including tumor vaccines, T-cell checkpoint blockade therapies, bispecific T-cell engagers, and adoptive cell therapies, all of which have demonstrated less clinical activity when used as monotherapies for prostate cancer. Finally, we anticipate that the sequence of these therapies will be important, timing the use of immune therapies to complement the effects of radiation therapy on the tumor immune microenvironment, to significantly improve the treatment and outcome for patients with advanced prostate cancer.

## Figures and Tables

**Table 1:** Physical characteristics of radionuclides commonly used in brachytherapy and targeted radionuclide therapy for prostate cancer.

Brachytherapy			
Radionuclide	Emission	Half-Life	Mean Energy (MeV)
Palladium-103	beta	17 days	0.021
Iodine-125	gamma	60 days	0.035
Iridium-192	gamma	74 days	0.355
Cesium-131	X-ray	9.7 days	0.029
**Targeted radionuclide therapy**			
Bismuth-213	alpha	0.03 days	8.37
Actinium-225	alpha	10 days	6
Radium-223	alpha	11.43 days	5.64
Strontium-89	beta	50.5 days	1.46
Samarium-153	beta	1.95 days	0.80
Gallium-68	beta	68 min	1.83
Lutetium-177	beta	6.7 days	0.497
Yttrium-90	beta	64.1 h	2.3

**Table 2:** Clinical trials using targeted radiation therapy in combination with immune-based treatments for prostate cancer.

Combination Immunotherapy		Phase	Number Enrolled	Study Title	NCT Trial No.	Results/Comments	Reference
**External Beam Radiation Therapy**							
Aglatimagene besadenovec + valacyclovir		III	711	Phase III study of Prostatak^®^ immunotherapy with standard radiation therapy for localized prostate cancer	NCT01436968		
Dendritic cells		I	5	Serial assessment of lymphocytes and apoptosis in the prostate during coordinated intraprostatic dendritic cell injection and radiotherapy	N/A		[123]
Dendritic cell-loaded vaccine (DCVAC)		II	62	Phase II study of DCVAC/PCA after primary radiotherapy for patients with high- risk localized prostate cancer	NCT02107430		
PSA-based vaccine + cryotherapy		I	45	A phase I feasibility study of an intraprostatic PSA- based vaccine in men with prostate cancer with local failure following radiotherapy or cryotherapy or clinical progression on androgen deprivation therapy in the absence of local definitive therapy	NCT00096551		
rvPSA vaccine		II	48	PSA-based vaccine and radiotherapy to treat localized prostate cancer	NCT00005916		[122]
Pembrolizumab and SD-101 (TLR9 antagonist), and androgen deprivation		II	42	Pembrolizumab +/− SD-101 in hormone-naïve oligometastatic prostate cancer with rt and iADT	NCT03007732		
Sipuleucel-T		II	51	Sipuleucel-t with or without radiation therapy in treating patients with hormone-resistant metastatic prostate cancer	NCT01807065	RT did not enhance the humoral and cellular responses associated with sipuleucel-T therapy	[121]
Avelumab, utomilumab, and anti-OX40 antibody PF-04518600		I/II	173	Avelumab, utomilumab, anti-ox40 antibody PF-04518600, and radiation therapy in treating patients with advanced malignancies	NCT03217747		
Sipuleucel-T		Observational	20	A multicenter trial enrolling men with advanced prostate cancer who are to receive combination radiation and sipuleucel-T	NCT02232230		
**Stereotactic radiation therapy**							
NKTR-214 (CD122- based immuunocytokine) and nivolumab		I	21	Platform study for prostate researching translational endpoints correlated to response to inform use of novel combinations	NCT03835533		
Durvalumab		II	96	Prostate cancer with oligometastatic relapse: combining stereotactic ablative radiotherapy and durvalumab (MEDI4736)	NCT03795207		
M9241 (IL-12-based immunocytokine)		II	65	T-cell clonality after stereotactic body radiation therapy alone and in combination with the immunocytokine m9241 in localized high- and intermediate-risk prostate cancer treated with androgen deprivation therapy	NCT05361798		
Sipuleucel-T		II	20	Sipuleucel-T and stereotactic ablative body radiation (SABR) for metastatic castrate-resistant prostate cancer (mCRPC)	NCT01818986		
**Brachytherapy**							
Nivolumab		I/II	44	Combination of nivolumab immunotherapy with radiation therapy and androgen deprivation therapy	NCT03543189		
**Targeted radionuclide therapy**							
**Combination Immunothe-rapy**	**Targeted radio-therapy**	**Phase**	**Number enrolled**	**Study title**	**NCT trial no.**	**Results/Comments**	**Reference**
Avelumab	Ra-223	I/II	24	Radiation medication (radium-223 dichloride) versus radium-223 dichloride plus radiation enhancing medication (M3814) versus radium-223 dichloride plus M3814 plus avelumab (a type of immunotherapy) for advanced prostate cancer not responsive to hormonal therapy	NCT04071236		
Atezolizumab	Ra-223	Ib	45	Safety and tolerability of atezolizumab (ATZ) in combination with radium-223 dichloride (Ra-223) in metastatic castrate-resistant prostate cancer (CRPC) progressed following treatment with an androgen pathway inhibitor	NCT02814669		[124]
Pembrolizu-mab	Ra-223	II	45	A randomized, phase II study evaluating the addition of pembrolizumab (MK-3475) to radium-223 in metastatic castration-resistant prostate cancer (mCRPC)	NCT03093428		
Nivolumab	Ra-223	I/II	36	Study of nivolumab in combination with radium-223 in men with metastatic castration-resistant prostate cancer	NCT04109729		
Sipuleucel-T	Ra-223	II	36	Study of sipuleucel-T with or without radium-223 in men with asymptomatic or minimally symptomatic bone-mCRPC	NCT02463799	Possibility of greater clinical activity with the combination of sipuleucel-T and radium-223; warranted further larger trial to confirm	[125]
PSA/TRICOM vaccine	153-Sm	II	44	^153^Sm-EDTMP with or without a PSA/TRICOM vaccine to treat men with androgen-insensitive prostate cancer	NCT00450619	^153^Sm-EDTMP in combination with PSA-TRICOM appears to lead to clinically meaningful improvement in PFS and is associated with trends to improved PSA declines and PSA-specific T-cell responses compared with ^153^Sm-EDTMP alone	[126]
Pembrolizumab	^225^Ac-J591	I/II	76	Maximizing responses to anti-PD1 immunotherapy with PSMA-targeted alpha therapy in mCRPC	NCT04946370		
Pembrolizumab	^177^Lu-PSMA	I/II	37	PRINCE (PSMA-lutetium radionuclide therapy and immunotherapy in prostate cancer)	NCT03658447		
Pembrolizumab	^177^Lu-PSMA-617	I	43	^177^Lu-PSMA-617 and pembrolizumab in treating patients with metastatic castration-resistant prostate cancer	NCT03805594		
